# Human dispersal into East Eurasia: ancient genome insights and the need for research on physiological adaptations

**DOI:** 10.1186/s40101-024-00382-3

**Published:** 2025-02-14

**Authors:** Steven Abood, Hiroki Oota

**Affiliations:** Department of Biological Sciences, Graduate School of Science, 7-3-1 Hongo, Bunkyo-ku, Tokyo, 113-0033 Japan

**Keywords:** Multiregional Evolution model, Out of Africa model, East Asian migration, Cold adaptation, Obesity

## Abstract

Humans have long pondered their genesis. The answer to the great question of where *Homo sapiens* come from has evolved in conjunction with biotechnologies that have allowed us to more brightly illuminate our distant past. The “Multiregional Evolution” model was once the hegemonic theory of *Homo sapiens* origins, but in the last 30 years, it has been supplanted by the “Out of Africa” model. Here, we review the major findings that have resulted in this paradigmatic shift. These include hominin brain expansion, classical insight from the mitochondrial genome (mtDNA) regarding the timing of the divergence point between Africans and non-Africans, and next-generation sequencing (NGS) of the Neanderthal and Denisovan genomes. These findings largely bolstered the “Out of Africa” model, although they also revealed a small degree of introgression of the Neanderthal and Denisovan genomes into those of non-African *Homo sapiens*. We also review paleogenomic studies for which migration route, north or south, early migrants to East Eurasia most likely traversed. Whichever route was taken, the migrants moved to higher latitudes, which necessitated adaptation for lower light conditions, colder clines, and pro-adipogenic mechanisms to counteract food scarcity. Further genetic and epigenetic investigations of these physiological adaptations constitute an integral aspect of the story of human origins and human migration to East Asia.

## The revised history of *Homo sapiens* over the past 30 years

Modern humans (*Homo sapiens*) originated in Africa approximately 300,000 years ago, with a group that left Africa around 60,000 years ago eventually dispersing across the earth [1, 2]. The hypothesis that *Homo sapiens* originated in Africa, known as the “Out of Africa” model, is now widely accepted [3, 4]. However, until the early 1990s, this hypothesis was just one of competing models. The alternative was the “Multiregional Evolution” model, which was, in fact, more widely supported [5, 6].

The dominant scientific consensus has undergone a dramatic shift over the past 30 years. Strong support for the “Out of Africa” model has emerged from both modern human genome data collected globally and ancient genome data, including those of Neanderthals [7–9]. The extensive genome-based analyses conducted thus far overwhelmingly support the African origin of modern humans, making it highly unlikely that this theory will be overturned.

**Fig. 1:**
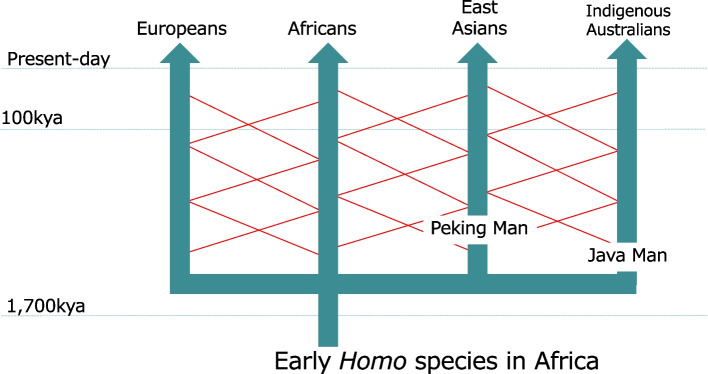
The Multiregional Evolution hypothesis

The “Multiregional Evolution” hypothesis proposes that the various human populations currently inhabiting different regions—such as Europeans, Africans, East Asians, and Indigenous Australians—each evolved independently into *Homo sapiens* within their respective regions (Fig. 1). Approximately, 1.7 million years ago, *Homo erectus* was the first hominin species to leave Africa, dispersing across the Eurasian continent [10]. Fossils of *Homo erectus*, such as Peking Man and Java Man, have been discovered in East and Southeast Asia, indicating that this species reached the easternmost parts of the continent [11, 12]. According to the “Multiregional Evolution” hypothesis, modern East Asians would be direct descendants of Peking Man, while modern Indigenous Australians and Southeast Asians would be direct descendants of Java Man.

However, the “Multiregional Evolution” hypothesis, which was primarily based on fossil evidence, presents significant logical inconsistencies, particularly in regard to the evolution of the brain. Early humans, such as *Homo erectus*, had a brain capacity ranging from approximately 700 to 1000 cubic centimeters (cc), while modern humans possess an average brain capacity of about 1350 cc—nearly double that of *Homo erectus* [13, 14]. It is unlikely that this substantial increase in brain size can be attributed to environmental factors or random chance alone. The brain expansion that so profoundly distinguished us from our ancestors likely resulted from a genetic mutation of great import that relaxed the constraints on brain development. Yet, the probability of such a mutation occurring independently in multiple geographical regions in a species is exceedingly low. Given the well-understood mutation rates of DNA, it is nearly impossible for a mutation leading to increased brain size to have occurred independently in multiple regions over a span of 1.5 million years. This represents a critical flaw in the “Multiregional Evolution” hypothesis.

To address this logical inconsistency, an additional explanation was proposed—namely, “frequent interbreeding between populations.” According to this scenario, a genetic mutation that led to an increase in brain size would have occurred once within a specific population, and this mutation would have spread to other regions through subsequent interbreeding. The multiple diagonal lines drawn between the vertical arrows in Figure 1 represent this hypothesized frequent hybridization. However, this explanation is also highly implausible. In the Paleolithic period, with no airplanes or cars, it is difficult to envision such extensive intercontinental interactions between populations occurring over a million years ago.

**Fig. 2:**
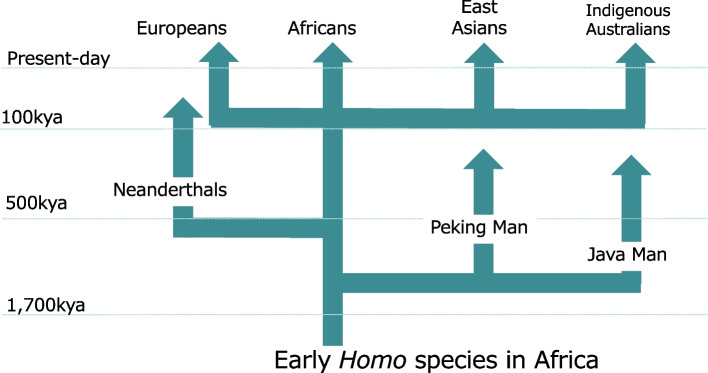
The Out of Africa hypothesis

In contrast, the “Out of Africa” theory suggests that a new species with a larger brain emerged in Africa and subsequently dispersed across the earth (Fig. 2). This theory explains why all modern humans, regardless of geographic location, possess large brains. Specifically, this theory proposes that a new hominin species with an expanded brain size originated on the African continent and later migrated out of Africa, becoming the direct ancestors of all modern humans. While this hypothesis was initially proposed by fossil anthropologists, molecular biologists played a crucial role in its validation [3]. By the 1980s, advancements in DNA technology allowed researchers to rigorously test the “Multiregional Evolution” hypothesis against the “Out of Africa” hypothesis at the molecular level. One of the pioneers in this field, Allan C. Wilson, along with his research team, examined the mitochondrial genome (mtDNA), which is maternally inherited [15]. They collected human samples from individuals around the world (*n* = 147) and utilized “restriction enzymes” to cut mtDNA at specific sequences, resulting in distinct cut patterns. These patterns, known as “haplotypes,” were utilized to classify the mtDNA and construct a phylogenetic tree, estimating the evolutionary relationships among the haplotypes. Their analysis revealed that the mtDNA of all 147 individuals could be traced back to an African woman. Furthermore, by calculating the divergence point at which African and non-African clusters separated on the mtDNA phylogenetic tree, they estimated that this event occurred approximately 200,000 years ago [15].

This divergence time was estimated by leveraging the molecular clock properties of DNA. A molecular clock refers to the consistent rate at which DNA sequences undergo mutations, resulting in clock-like changes over time. Both the “Multiregional Evolution” and the “Out of Africa” hypotheses share the same starting point in that they both postulate that the origin of modern humans lies in Africa. Therefore, evidence indicating that the ancestors of all modern humans originated in Africa supports both theories to some extent. However, the discovery that the common ancestor of all modern humans was a single woman who lived approximately 200,000 years ago strongly favors the “Out of Africa” hypothesis [16]. If the “Multiregional Evolution” hypothesis were correct, the common ancestor would have been much older, dating back 1 to 2 million years.

As the so-called mitochondrial Eve hypothesis gained wider recognition, further analysis of nuclear DNA from autosomes and sex chromosomes also produced results that supported a single origin in Africa [17–19]. Consequently, the credibility of the “Out of Africa” hypothesis steadily grew among geneticists. However, it was the subsequent publication of the Neanderthal mtDNA sequence that solidified the “Out of Africa” hypothesis as the prevailing consensus among researchers studying human history [20].

Even if the “Out of Africa” hypothesis is correct, several questions remain. For instance, the ancestors of Neanderthals are known to have left Africa earlier than *Homo sapiens* and subsequently evolved in Europe. If the “Multiregional Evolution” hypothesis was accurate, it would be reasonable to consider Neanderthals as the direct ancestors of modern Europeans. However, under the “Out of Africa” hypothesis, Neanderthals would have been replaced by *Homo sapiens*, implying that Neanderthals are not the direct ancestors of modern Europeans. Given that Neanderthals and *Homo sapiens* were much more similar to each other than to any other earlier hominins, their genetic relationship became a critical area of investigation.

## Paleogenomics revealed Neanderthal extinction and hybridization with *Homo sapiens*

The most effective method to examine whether Neanderthals went extinct is through whole-genome analysis of DNA extracted from Neanderthal bones. However, until the twenty-first century, it was technically almost impossible to fully sequence the Neanderthal genome. Instead of full genome sequencing, researchers focused on sequencing a part of Neanderthal mtDNA and comparing it with those previously studied in modern humans worldwide [20].

Svante Pääbo was a pioneer in the field of ancient DNA analysis and was later awarded the Nobel Prize in Physiology or Medicine in 2022 for his groundbreaking work in decoding the Neanderthal genome as well as the genome of the Denisovans, another archaic hominin species. Before achieving the full sequencing of these genomes, Pääbo’s research team successfully determined a sequence of approximately 380 base pairs from the D-loop region of Neanderthal mtDNA [20]. When this sequence was compared with those of modern humans to construct a molecular phylogenetic tree, it was found that all modern humans form a single cluster, while the Neanderthal branch is positioned outside of this cluster [20]. The topology of this phylogenetic tree revealed that the Neanderthal mtDNA sequence fell outside the range of mtDNA diversity observed in modern humans. The significant difference in the Neanderthal mtDNA sequence led to the conclusion that Neanderthals and modern humans were biologically distinct species. Since no modern humans were found to carry Neanderthal (or Neanderthal-like) mtDNA sequences, many researchers believed that Neanderthals likely went extinct.

In 2003, the completion of the Human Genome Project was declared, accelerating the development of technologies that enabled personal genome analysis. Several sequencing technologies, collectively known as next-generation sequencers (NGS), which operate on principles entirely different from the earlier Sanger method, were developed. These NGS technologies were rapidly adopted, significantly advancing the analysis of the Neanderthal genome. In 2010, the draft sequence of the entire Neanderthal genome was published. Calculations based on this genome sequence indicated that Neanderthals and modern humans diverged between 500,000 and 600,000 years ago [21]. This conclusion was consistent with the results of the mtDNA analysis by Krings et al. [20]. However, nuclear DNA revealed insights that could not be discerned from mtDNA alone. After their divergence, Neanderthals and modern humans encountered each other again and interbred in West Asia approximately 50,000 to 60,000 years ago [21].

Analysis of the Neanderthal genome revealed that 1 to 4% of the genome in modern humans living outside Africa is derived from Neanderthals [21], providing evidence that interbreeding between the two species occurred in the past, and that they produced offspring. Since NGS has a higher error rate than Sanger sequencing, there were concerns that the draft sequence from Green et al. (2010) might have included some sequencing errors. However, the complete sequence, which was read over 50 times, was published, confirming the hybridization between the two species [22]. Thus, the “Out of Africa” hypothesis proved to be more complex than initially thought. In addition to Neanderthals, the Denisovans, another archaic hominin group, were discovered through genome analysis of a finger bone, revealing that they too had interbred with *Homo sapiens* [23]. Despite these discoveries of hybridization, the vast majority of the modern human genome still originates from Africa, leaving the “Out of Africa” hypothesis fundamentally intact.

## The dispersal of *Homo sapiens* into eastern Eurasia

Researchers in archeology and anthropology in East Asia have traditionally interpreted human history in the region through the lens of the “Multiregional Evolution” hypothesis. However, over the past 30 years, the widespread acceptance of the “Out of Africa” theory has led to a significant paradigm shift, compelling East Asian archeology and anthropology to reevaluate the history of *Homo sapiens*’ spread to the eastern end of the Eurasian continent. This reconsideration is particularly important because East Eurasia serves as the ancestral homeland for Oceanian peoples and Native Americans.

Following the declaration of the completion of the Human Genome Project in 2003, an international initiative known as the “HapMap Project” was launched, with its first paper published in 2005. The project aimed to establish genetic evidence related to heritable diseases and drug responses [24] Utilizing the approximately 3 billion base pairs of the human reference genome, the HapMap Project sought to create a comprehensive database of chromosomal locations and population-specific frequencies of genetic variations across African, European, and East Asian populations by comparing these variations with the reference genome.

The variations examined in the project were primarily single-nucleotide polymorphisms (SNPs). The project published data showing the frequency of specific SNPs in each population, which were made available on a public website. Although the main purpose of this project was the medical and pharmaceutical use of such SNPs as markers, a key finding in the context of human history was that African populations exhibited significantly higher genetic diversity compared to non-African populations [24, 25]. This finding aligns perfectly with the scenario of modern humans originating in Africa and subsequently dispersing across the globe after leaving Africa. Thus, while the data on human genome diversity was initially collected for projects focused on genomic medicine and drug discovery, the analysis of this data provided strong support for the “Out of Africa” hypothesis for the origin of modern humans.

The HapMap Project employed a technique called “genotyping” to examine specific locations of mutations. Following this, another international initiative, the “1000 Genomes Project,” was launched with the goal of sequencing the entire genomes of 1000 individuals, which was completed in 2015 [26]. Although this project was also primarily conducted for medical and pharmacological purposes, the data analysis further reinforced the “Out of Africa” theory.

A study on the genome diversity of East Asian populations was published which further illuminated the migration patterns of humans out of Africa to East Asia [27]. The analysis of the SNP data revealed a phylogenetic tree in which the Indian population branched off first from the African population, followed by the Indonesian and other Southeast Asian populations, then the Thailand population, and finally the Chinese, Korean, and Japanese populations. The topology of this phylogenetic tree supports a scenario in which *Homo sapiens* migrated out of Africa, passed through the Indian subcontinent, reached Southeast Asia, and then moved northward, eventually giving rise to the modern East Asian populations. However, this result was not initially intuitive from East Asian Paleolithic archeological evidence.

**Fig. 3:**
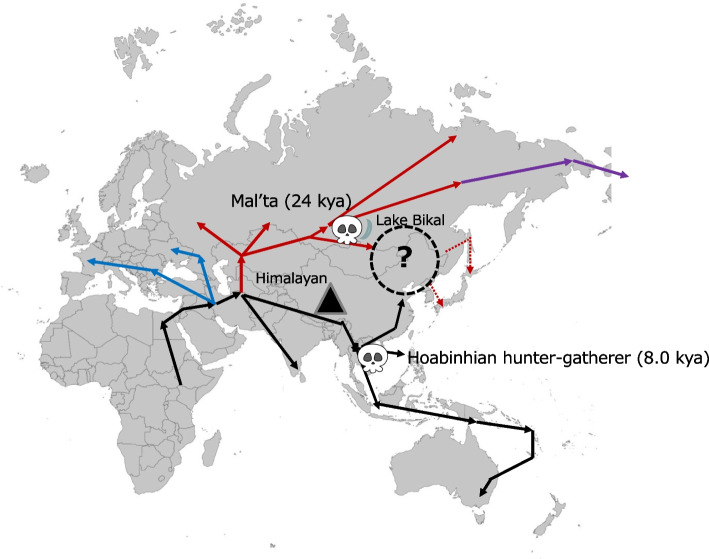
The dispersal of *Homo sapiens* into eastern Eurasia, modified from Kaifu, Izuho, and Goebel (2015)

Archeological evidence suggests two potential dispersal routes for *Homo sapiens* along the eastern side of the Eurasian continent following their departure from Africa: the northern route and the southern route (Fig. 3). Considering the Himalayas as a natural boundary, we define these routes as the “northern route,” which passes north of the Himalayas, and the “southern route,” which passes south of the Himalayas, extending toward the eastern edge of the Eurasian continent [28]. Microblades, a distinctive type of stone tool, are frequently found around Lake Baikal, a region believed to be a key link in the northern route. Notably, microblades are absent south of the Qinling–Huaihe Line, which delineates North and South China [29]. However, the Pan-Asia SNP Consortium (2009) presented genetic evidence supporting only the southern route [27]. Thus, there is a discrepancy between the migration routes indicated by genomic data and those suggested by archeological evidence.

## Ancient genome analysis of *Homo sapiens* in eastern Eurasia

Svante Pääbo’s group extracted DNA from the bones of a modern human who lived approximately 40,000 years ago, discovered in Tianyuan Cave near Beijing. They analyzed the mtDNA and chromosome 21, the smallest of the 22 autosomes [30]. The analysis revealed that the Tianyuan individual belonged to a population ancestral to many modern East Asians and Native Americans. This ancestral group diverged after the split between the ancestors of East Asians and Europeans. The study highlighted that the ancestors of Native Americans originated from the East Asian lineage after their separation from Europeans. The same group later published a study which included the whole genome of the Tianyuan individual [31]. This study analyzed the data to explore the relationships between modern Europeans, East Asians, and Native Americans, but the issue concerning the northern versus southern migration routes of East Eurasians was beyond the scope of the study.

The earliest evidence of *Homo sapiens* activity in the Japanese archipelago is found in stone tools dating back approximately 38,000 years [32]. However, due to the volcanic nature of the Honshu and Hokkaido islands, very few human remains have been discovered, and the DNA from these remains is highly fragmented, making it unsuitable for ancient genome analysis. In contrast, numerous human bones from the Jomon period, which began around 16,000 years ago, have been excavated, particularly from shell mounds. These remains have been thoroughly studied and carefully preserved by archeologists and anthropologists.

Whether the Jomon people are direct descendants of the Paleolithic inhabitants of the Japanese archipelago remains a subject that requires further investigation. If the Jomon people are indeed direct descendants, their genome sequence data could provide crucial evidence to determine whether modern East Asians emerged from the hybridization of populations from both the northern and southern routes. McColl et al. (2018) reported the draft sequence of the entire Jomon genome [33]. The Jomon individual analyzed in this study, excavated from the Ikawazu Shell Mound in Aichi Prefecture, shared many genetic variants with a hunter-gatherer from the Hoabinhian culture, who lived approximately 8000 years ago in what is now Laos, Southeast Asia (Fig. 3). This finding strongly supports the “dual structure model” proposed by Hanihara (1991), which suggests that the ancestors of the late-Paleolithic inhabitants of the Japanese archipelago were “Proto-Asians” who had lived in Sundaland (present-day Southeast Asia) for over 30,000 years [34].

Previously, a genome sequence was reported from human remains from approximately 24,000 years ago discovered at the Malta site near Lake Baikal [35]. This study revealed that modern Northeast Asians and Native Americans received significant gene inflow from this lineage. This suggests that Northeast Asians and Native Americans originated by interbreeding between people from both the southern and northern routes. The Mal’ta individual can be considered representative of the northern route.

Gakuhari et al. (2020) analyzed the genome sequence data of the Ikawazu Jomon individual within the context of the southern versus northern routes [36]. The results indicated that all modern East Asians, Northeast Asians, and Native Americans, including this Jomon individual, are descendants of populations that reached the eastern side of the Eurasian continent via the southern route. When Gakuhari et al. (2020) constructed a phylogenetic tree using genome sequence data from individuals who currently or historically lived on the eastern side of the Eurasian continent, they found that a cluster of ancient human remains near Lake Baikal, including the Mal’ta individual, diverged from another cluster that encompassed all modern East Asians, Northeast Asians, and Native Americans (Fig. 1c in Gakuhari et al., 2020) [36]. The ancient remains of the Hoabinhian hunter-gatherer (approximately 8000 years ago) and the modern hunter-gatherers of the Andaman Islands formed a cluster, with the next branch diverging to include the Tianyuan individual (approximately 40,000 years ago). Interestingly, the subsequent divergence was that of the Kusunda people, a minority ethnic group in Nepal, followed by the Ikawazu Jomon individual. All modern and ancient East Asian, Northeast Asian, and American populations were positioned within the branch that includes the Ikawazu Jomon individual (see Fig. 1c in Gakuhari et al., 2020) [36]. This figure suggests that the Jomon people are direct descendants of the basal population of the eastern Eurasian continent.

As shown by Raghavan et al. (2014), the genome of the Mal’ta individual had a significant gene flow into Northeast Asians and Native Americans [35]. The genetic influence of people from the northern route on Northeast Asians and Native Americans merely replicated previous findings in Gakuhari et al. (2020) [36]. However, the fact that no evidence of gene flow from Mal’ta was detected in the genome of the Ikawazu Jomon individual was a novel discovery. To what extent did the genome of the northern route influence the Jomon people? In the genome of the Ikawazu Jomon individual, no evidence of gene flow from the northern route was detected. Sufficient DNA was extracted from one of the two Jomon skeletons excavated from the Funadomari site in Hokkaido, allowing for the determination of a complete genome sequence [37]. The Funadomari Jomon genome sequence had a high depth of coverage, and analysis revealed a remarkable genetic similarity to the Ikawazu Jomon. Including this Funadomari Jomon sequence, genome sequence data from approximately 10 Jomon individuals have been registered in public databases and are available for analysis [33, 37, 38]. To date, the conclusion remains unchanged. However, not all Jomon genome sequences analyzed so far have undergone the type of southern versus northern route-focused analysis presented in Gakuhari et al. (2020) [36]. Conducting further analysis on this issue remains a task for the future.

## The importance of understanding physiological adaptations

Around 60,000 years ago, as *Homo sapiens* began dispersing from Africa, the Earth was in the last glacial period. The Tianyuan individual dates back to approximately 40,000 years ago, and the earliest evidence of *Homo sapiens* activity in the Japanese archipelago, indicated by stone tools, dates to around 38,000 years ago [31, 32]. Thus, the spread of *Homo sapiens* across Eurasia also occurred during the Ice Age. People who traveled the southern route from Southeast Asia to the north needed to adapt to decreasing sunlight and colder climates. Conversely, the risk of pathogens prevalent in warmer regions may have diminished as they moved northward. Those who traveled the northern route were able to migrate southward to certain parts of East Asia, but they did not spread further south, likely because they had not developed the same level of pathogen resistance as those who took the southern route [39].

Whether they followed the northern or southern route, the migration of *Homo sapiens* into eastern Eurasia involved moving from lower to higher latitudes, requiring adaptation to colder climates and reduced sunlight [40]. Akiyama et al. (2017) investigated the relationship between physiological variations in response to light stimuli and *PER2* gene polymorphisms in experimental conditions [41]. They found that three common haplotypes of *PER2* accounted for more than 96% of the participants’ chromosomes, with one of these haplotypes exhibiting significantly lower sensitivity to light stimuli (*P* < 0.05). Individuals homozygous for the low-sensitivity *PER2* haplotype showed significantly lower melatonin suppression rates (*P* < 0.05) compared to heterozygous individuals. This finding suggests that physiological variations in light sensitivity are closely associated with *PER2* polymorphisms. When global haplotype frequencies were compared, the low-sensitivity haplotype was found to be more prevalent in Africans than in non-Africans and was positioned at the root of the phylogenetic tree. This indicates that the low-sensitivity haplotype is ancestral, while the high-sensitivity haplotypes are derived. Consequently, it is hypothesized that the high-sensitivity haplotypes spread globally after modern humans migrated out of Africa.

In addition to light sensitivity alleles, a plethora of cold adaptations may also have been selected for. These include genotypes related to isolative adaptation, where skin temperature changes in response to cold but core body temperature is maintained; hypothermic adaptation, in which a person exhibits lower core body temperature; isolative hypothermic adaptation, in which a person exhibits both a decrease in skin temperature and lower core body temperature; and metabolic adaptation, in which a person possesses enhanced thermogenesis [42].

Nishimura et al. (2017) investigated uncoupling protein 1 (UCP1), which inhibits ATP synthesis on the mitochondrial membrane of brown adipose tissue (BAT) and promotes thermogenesis [43]. They demonstrated that the degree of non-shivering thermogenesis (NST) in healthy individuals within an artificial climate chamber varied significantly according to *UCP1* genotype. The frequency of the haplotype associated with the highest NST was found to be significantly correlated with latitude and environmental temperature. This finding provides the first evidence that *UCP1* genotype influences the efficiency of NST in humans, supporting the hypothesis that the *UCP1* gene has played a role in cold adaptation throughout human evolutionary history.

The *UCP1* SNPs Nishimura et al. (2017) identified as conferring significantly high levels of thermogenesis are strongly linked to the neighbor gene *ELMOD2*, which may confer antiviral benefits, as elevation in body temperature improves antiviral immunity [43]. Nishimura et al. (2017) hypothesizes that the *UCP1* gene and the *ELMOD2* gene were important for the adaptive utilities of cold adaptation and viral resistance as humans migrated away from the equator [43].

Ishida et al. (2024) conducted a study which investigated the associations between physiological traits induced by cold exposure in 399 healthy Japanese and 11 SNPs at 6 loci that are thought to be under cold adaptation [44]. They measured the BAT activity for the volunteers, performed genotyping for them, and tested associations between the SNP genotypes and BAT activity or other related traits. Although these findings did not survive multiple test comparisons, they showed two SNPs in *LEPR* (rs1022981 and rs12405556) that were associated with higher BAT activity [44]. Thus, cold adaptation in East Asians remains a subject of intrigue, and active research has been conducted combining physiological experiments and population genetic methods.

Some studies have argued that present-day East Eurasians, a portion of whose ancestors spent thousands of years in frigid Siberia adapting to the cold climate, have higher degrees of obesity resistance when compared to Europeans and Africans [45–47]. Cold adaptive genetic variations in the magnitude of adaptive thermogenesis may account for these differences. Populations whose ancestors were highly cold adapted generally possess higher basal metabolic rates [48, 49]. Although Native Americans (including Pima Indians), Pacific Islanders, and East Asians have the same ancestral lineage and could share cold-adapted genetic traits, only East Asians appear to have high metabolic rates that confer obesity resistance [36, 50, 51]. The reason why such differences occurred remains an issue for future study and requires further research [52].

An allele at the T-box transcription factor 15/mitochondrial tryptophan tRNA synthetase 2 (*TBX15*/*WARS2*) locus potentially mediates thermogenic responses by affecting brown and beige adipocyte differentiation [53]. The *TBX15/WARS2* allele is found in the highest frequencies in present-day East Asians and the Inuits, lower frequencies in Europeans, and the lowest frequencies in Africans [54]. Racimo et al. (2017) concludes that the allele was likely introgressed from cold-adapted Denisovans [54]. Traditionally, environmental adaptations have been analyzed using the genome information of modern populations, but in the future, by analyzing the genomes of ancient populations, it will be possible to trace older environmental adaptations.

Ocobock et al. (2022) found evidence that BAT functions as a cold adaptation in a sub-arctic Finland population [55]. In this population, areas of the body with brown adipose tissue exhibited an 8.7% increase in metabolic rates, preferentially metabolized fatty acids, and maintained relatively warmer body surface, than areas without brown adipose tissue [55]. Sun et al. (2019) generated *TBX15* knockout mice and demonstrated that upon cold exposure or induction by β3 adrenergic agonist CL316243, adipocyte browning was significantly impaired in the knockouts [53]. They also utilized chromatin immunoprecipitation (ChIP) to find that TBX15 bound directly to a key region in the *Prdm16* promoter, indicating that TBX15 regulates transcription of *Prdm16*, the master gene for adipocyte thermogenesis and browning, and found that *TBX15* knockout mice gained more body weight in response to high-fat diets compared to controls [53]. Ishida et al. (2024), however, failed to find associations between *TBX15* SNPs and BAT thermogenesis [44]. Ishida et al. (2024) speculated that instead of brown adipose tissue thermogenesis, the *TBX15* loci may instead be related to body fat distribution and facial morphology, physiological traits that are also important for cold adaptation [44].

Previous researchers have found evidence that the *TBX15* locus is associated with facial morphology including the morphology of the outer ear [56, 57]. Bonfante et al. (2021) performed a genome-wide association study of more than 6000 individuals and found that *TBX15* was associated with lip thickness [58]. These facial morphological features could all plausibly constitute cold adaptations which helped ameliorate the physiological hardships of the exodus to higher latitudes.

## High-altitude adaptations and Denisovan introgressions

High-altitude adaptations allowed modern humans to migrate to and survive in mountainous regions and thus constitute a topic of great import in the story of the East Eurasians and their dispersal to extreme niches across the earth. High-altitude variants of *EPAS1* which resulted in lower hemoglobin and lower blood viscosity thus allowed our ancestors to colonize high-altitude regions [59].

Arima et al. (2024) found significant sex differences in *EPAS1* rs13419896 with a greater proportion of males carrying nonadaptive gene variants for high-altitude adaptation [60]. Arima et al. (2024) also found that higher incidences of obesity and potential gene flow in Tibetan highlanders living in the Mustang plateau may be responsible for higher rates of Monge’s disease compared to other populations living in the Tibetan plateau [60].

Further investigations into high-altitude genomic adaptations in the Tibetan Plateau were conducted by Ye et al. (2024) [61]. The investigators hypothesized that various traits related to oxygen delivery and the response to high-altitude hypoxia would predict lifetime reproductive success and show genomic associations in ethnic Tibetan women living at high altitude. They found that stabilizing selection favoring a modal hemoglobin concentration and directional selection favoring high oxygen saturation of hemoglobin together increased oxygen content and correlated with high lifetime reproductive success. Oxygen transport in women who had more live births was facilitated by a lower hypoxic heart rate response, wider left ventricles, and more effective flow-mediated vasodilation, which allowed the women to increase oxygen transport through the facilitation of large blood volumes from the heart without raising blood pressure [61].

What are the origins of such beneficial variants of *EPAS1*? A seminal paper by Huerta-Sánchez et al. (2014) relayed the startling conclusion that the origins of *EPAS1* in our ancestors were not due to a mutation but from introgression from an entirely different ancient hominin species, the Denisovans [62].

The Denisovans are thought to have gone extinct soon after they mated with the ancestors of Europeans and Asians between 30,000 and 40,000 years ago. The high-altitude *EPAS1* variant spread extremely rapidly among Tibetans in the course of approximately 3000 years. Huerta-Sanchez et al. (2014) sequenced *EPAS1* in 40 Tibetans and 40 Han Chinese who were once part of the same population before it split between 2750 and 5500 years ago. No matches of a 5-SNP motif (AGGAA) of the *EPAS1* high-altitude variant common in Tibetans were found by subsequently searching global genomes through the 1000 Genomes Project except for a single southern Han Chinese and a single Beijing Han Chinese individual. Huerta-Sánchez et al. (2014) did find a match however when they compared the high-altitude *EPAS1* variant to the ancient hominin finger bone found in Denisova Cave in the Altai Mountains of Siberia. Since the Denisova Cave is not in high altitude, the high-altitude *EPAS1* variant may have originally served as a cold adaptation, helping Denisovans dilate their blood vessels in cold climates that tend to constrict blood vessels and raise blood pressure which can lead to cardiovascular pathologies, before being co-opted to enhance high-altitude survival and reproduction [62].

*EPAS1* is not the only gene positively selected for to augment the physiology of Tibetans who encounter the decrease in partial pressure of oxygen in high altitude which results in fewer oxygen molecules per breath. Moreover, low partial pressure of oxygen is not the only environmental challenge of high altitude. Inhabitants of high altitude also encounter increased ultraviolet radiation exposure, extremely low temperatures, extremely low absolute humidity, and limited food resources, which can affect the immune system and make organisms more susceptible to cancer, as well as various infectious and autoimmune diseases [62–64]. Yet, despite these physiological challenges, high-altitude regions have been inhabited throughout human history. It is now estimated that 500.3 million humans live at or over 1500 m in altitude, 81.6 million at or over 2500 m, and 14.4 million at or over 3500 m [63]. There are even some areas of human habitation over 4000 m in altitude [63]. Selection for several beneficial high-altitude genes has made survival and reproduction at these roofs of the world possible for *Homo sapiens*. Denisovan gene insertions have contributed significantly to this border adaptation.

## Adaptations from Neanderthal introgressions

As mentioned above, there is evidence of Neanderthal introgression into the *Homo sapiens* genome [21]. Most Neanderthal alleles were deleterious and were negatively selected against, but some facilitated human adaptation to novel environments [65]. Neanderthals possessed these adaptive traits since they were living in Eurasian environments for more than 400,000 years which gave them ample time to adapt. When a subset of modern humans spread out of Africa, likely around 70,000 years ago, they co-opted these Neanderthal traits through interbreeding with them, which allowed them to survive novel environmental conditions including reduced UV exposure resulting in less endogenously produced vitamin D and new pathogens [65–67].

East Asian populations possess approximately 12 to 20% more Neanderthal DNA than Europeans [68, 69], while 1 to 4% of the DNA of present-day non-African populations was inherited from Neanderthals [21, 65, 70]. It remains a matter of ongoing debate whether these differential amounts of Neanderthal DNA are due to multiple admixture events with Neanderthals after their divergence from Europeans, negative selection against deleterious Neanderthal alleles, or dilution of Neanderthal ancestry in Europeans by unadmixed populations [71–75].

The three *OAS* genes on Chromosome 12, *OAS1*, *OAS2*, and *OAS3*, are introgressed from Neanderthal DNA and constitute key components of the antiviral response of the innate immune system [76–78]. Interferons induce *OAS* gene expression and activate RNase L, leading to degradation of viral RNAs, thereby inhibiting viral protein synthesis [79]. The adaptive Neanderthal *OAS* haplotype is observed at approximately 30% frequency in European and South Asian populations and at 20% frequency in populations in East Asia and the Americas [77, 80]. Remarkably, the *OAS* haplotype at *OAS1* was recently shown to be protective against COVID-19 severity, hospitalization, and susceptibility [81]. Haplotypes at this locus have also shown variable expression responses to different flaviviruses [80]. Prior to these findings regarding *OAS1*, a well-known study showed that a gene cluster on chromosome 3 associated with the risk factor for severe symptoms after SARS-CoV-2 infection and hospitalization in present-day humans was of Neanderthal origin [81]. A subset of virus-interacting proteins that interact with coronaviruses show signals of positive selection within the last 25,000 years in East Asians, suggesting the existence of the selective pressure of an ancient coronavirus epidemic [82].

Turning next to metabolism, one study proposed that Neanderthal alleles in lipid catabolism genes have been targets of recent positive natural selection in Europeans [83]. A genome-wide association study performed on a large cohort of over 8000 Mexicans and Latin Americans identified a Neanderthal introgressed haplotype on gene *SLC16A11* associated with hepatic lipid metabolism that increased risk for type 2 diabetes by approximately 20% [84]. The haplotype is at lower frequency in East Asians than its approximately 50% frequency in Mexican populations and nearly absent in other populations [84]. The *TSHR* gene was also identified in Europeans as a locus resulting from Neanderthal adaptive introgression [84]. The *TSHR* gene encodes the thyroid-stimulating hormone receptor which binds thyrotropin resulting in the growth of the thyroid gland and facilitation of thyroid-related metabolic processes [84]. *TSHR* also plays important roles in adipocyte differentiation and lipolysis [85, 86].

The African exodus darkened the world for *Homo sapiens*, who encountered less UV-B irradiation, and thus less endogenous vitamin D production, than they had in their ancestral homeland. Lighter pigmentation could compensate for the diminished sun and allow more vitamin D production, essential for the health of a plethora of bodily processes. So, the migrants acquired a cadre of pigmentation genes from the Neanderthals, including *HYAL2*, *BNC2*, *POU2F3*, *THEM136*, *MC1R*, *OCA2*, *KRT71*, and *KRT80* [72, 76, 77, 87–89].

Out of 246 circadian genes, variants introgressed from Neanderthals increased the chronotype of morningness, the propensity to wake up early [66]. Morningness is consistent with adaptations to high latitude observed in other latitudes and is associated in humans with a shortened period of the circadian clock. Shortened circadian periods are useful for synchronization to the extended summer light periods of high latitudes, and selection for shorter circadian periods has resulted in latitudinal clines of decreasing period with increasing latitude in *Drosophila* populations. Therefore, the propensity of introgressed variants to increase morningness may indicate selection toward shortened circadian period in the populations living at high latitudes [66].

In addition to the aforementioned traits, two genome-wide association studies also found Neanderthal variants associated with height, heart rate, blood disorders, tobacco use, and mood disorders [90, 91].

## Mismatched genes

Genetic mutations that were advantageous in a certain area in the past may not only lose their benefits over time but may even become slightly deleterious as the environment changes. For instance, genes that may promote immune function in the bright light environment of equatorial Africa such as *TSPAN10* may contribute to the evolutionary mismatch of myopia in the lower light conditions migrants faced when they traversed to higher latitudes [92].

Another example of previously beneficial mutations which are hypothesized to have become deleterious in our modern environment is “thrifty genes,” genes which favor a pro-adipogenic phenotype. In environments where food was scarce, mutations that reduced energy consumption and stored it as fat were advantageous for survival. However, in the present, many of these mutations are often identified as contributing factors to obesity and its related diseases. Diseases linked to obesity such as type 2 diabetes, heart disease, breast cancer, asthma, osteoporosis, gout, and depression are rare or completely absent in some modern hunters and gatherer populations whose lifestyles mimic our hunting and gathering ancestors [93–95].

The selection for and maintenance of a larger body size that could support more adipose tissue may have allowed the genus *Homo* to metabolically rely on both adipose reserves and low caloric food such as plant underground storage organs [96]. Such low caloric food would return too low a rate of energy for a smaller animal that could not rely on fat stores while entering into the caloric deficit necessary to seek out such foods [96]. However, a corollary hypothesis is that an increase in high-energy density food acquisition, and a concomitant adipose tissue-storing thrifty phenotype, were the costs of the development of larger brains [97]. The *Homo sapiens* brain, a high-fat organ of approximately 33% lipid content, accounts for roughly 2% of a person’s body weight yet demands at least 20% of the body’s energy consumption [98]. So, to maintain the energy requirements of a larger brain necessary for a cognitive processing capacity that supports survival, especially in times of food scarcity, a genotype that favored the storage of adipose tissue reserves may have been selected for [97].

*Homo sapiens*, who dispersed during the ice age, likely carried thrifty gene mutations to some extent, enabling them to survive in harsh environments. While some experts advocate for anti-obesity drugs and even gene editing of thrifty genes to ameliorate the global obesity crisis, improved psychobiological methods to create and maintain habits regarding the elimination of obesogens, as well as lifestyle changes in the areas of food quality and quantity, timing of food intake, and caloric expenditure, constitute a safer and thus more prudent intervention.

## Conclusions

In the last 30 years, modern and ancient human genome data have garnered a paradigmatic shift from the “Multiregional Evolution” model to the “Out of Africa” model of human origins. The “Out of Africa” theory possesses significantly more explanatory power, as it accounts for the universality of modern human brain expansion. In addition, studies on mtDNA, nuclear DNA from autosomes and sex chromosomes, the Neanderthal mtDNA sequence, and the complete Neanderthal genome all point to our ancestors who lived in Africa. Although studies have found that Neanderthal and Denisovan hybridization with *Homo sapiens* occurred after the divergence of *Homo sapiens* from both of them, the vast majority of the modern human genome still originates from Africa, leaving the “Out of Africa” hypothesis fundamentally secure.

Two subsequent studies, the “HapMap Project,” examining primarily SNPs, and the “1000 Genomes Project,” examining whole-genome sequences, further bolstered the “Out of Africa” theory. As would be expected by the theory, diversity was found to decrease with further geographic distance away from Africa.

Further studies shed light on the peopling of East Asia, and supported a scenario in which *Homo sapiens* migrated out of Africa, eventually giving rise to the modern East Asian populations. Archeological evidence suggests two potential dispersal routes, a northern and a southern route, for these ancestors of modern East Eurasians. While the microblade evidence supports the northern route, the genetic evidence presented by the Pan-Asian SNP Consortium supports the southern route. This discrepancy was addressed by the genome analysis of the Jomon people, who are likely to be direct descendants of those who lived in the Japanese archipelago during the Paleolithic period. The results revealed that the Jomon, present-day East Asian, Northeast Asian populations, and Native Americans have genomes of the southern route, and that Northeast Asian and Native American populations have a large gene flow of the northern route, but the Jomon and present-day East Asian populations do not. However, only a few genome analyses have been reported on the theme of interbreeding between northern and south routes, and further analysis is required in the future. Whether the ancestors of modern East Asians followed the northern or southern route, the migration of *Homo sapiens* into eastern Eurasia involved moving from lower to higher latitudes, requiring adaptation to colder climates, diminished sunlight, and food scarcity. This likely led to adaptation for *PER2* gene polymorphisms which conferred variable sensitivities to light stimuli, cold adaptations including those involving *UCP1*, viral resistance conferred by polymorphisms of *ELMOD2*, an allele at the T-box transcription factor 15/mitochondrial tryptophan tRNA synthetase 2 (*TBX15*/*WARS2*) locus potentially mediating thermogenic responses by affecting brown and beige adipocyte differentiation, and adaptation for genes which confer a pro-adipogenic phenotype.

The examples summarized here are just a few among many reports of genetic polymorphisms related to physiological adaptation. *Homo sapiens* had to adapt physiologically to vastly different natural environments within an extremely short evolutionary time frame. Experimental studies with genomic analysis investigating these polymorphisms related to physiological adaptations are still limited, and thus, the pursuit of further such investigations are of the utmost importance and necessity in unraveling our human story.

It is reasonable to infer that physiological adaptations identified in the studies conducted to date are not solely based on genetic mutations, as the timeline of *Homo sapiens*’ spread is too recent for significant genetic changes to have accumulated. Therefore, physiological adaptations driven by epigenetic mechanisms, such as DNA methylation and acetylation that regulate gene expression, may be more significant than those based on genetic mutations. However, research into these mechanisms is still in its early stages. Understanding these adaptations is a future challenge, as our modern physiological characteristics are the result of *Homo sapiens*’ evolution, and their true essence can only be fully understood from an evolutionary perspective.

## Data Availability

Not applicable.

## Abbreviations

GWADS: Genome-wide allelic differentiation scan
mtDNA: Mitochondrial deoxyribonucleic acid
NGS: Next-generation sequencing
SNPs: Single-nucleotide polymorphisms
TBX15/WARS2: T-box transcription factor 15/mitochondrial tryptophan tRNA synthetase 2
UCP1: Uncoupling protein 1
